# Nursing Students' Participation and Perceptions of Teaching Effectiveness at Clinical Teaching Bases: A Cross‐Sectional Survey

**DOI:** 10.1002/nop2.70824

**Published:** 2026-10-06

**Authors:** Hao Zhang, Yuan Mei, Chun‐ling Zhang, Jie Liu, Xu‐jue Ma

**Affiliations:** ^1^ Department of Radiology Dianjiang People's Hospital of Chongqing Chongqing P. R. China; ^2^ Department of Hospital‐Acquired Infection Control Dianjiang People's Hospital of Chongqing Chongqing P. R. China; ^3^ Department of Medical Administration Dianjiang Health Commission of Chongqing Chongqing P. R. China; ^4^ Department of Scientific Research and Education Dianjiang People's Hospital of Chongqing Chongqing P. R. China; ^5^ Department of Orthopedics The First Affiliated Hospital of Chongqing Medical and Pharmaceutical College Chongqing P. R. China; ^6^ Department of Critical Care Medicine The First Affiliated Hospital of Chongqing Medical and Pharmaceutical College Chongqing P. R. China

**Keywords:** clinical practicum, nursing education, student engagement, student feedback, teaching effectiveness

## Abstract

**Aim:**

To assess nursing students' participation and perceptions of teaching effectiveness at clinical teaching bases and to identify the improvements students themselves prioritize.

**Design:**

A cross‐sectional, descriptive survey.

**Methods:**

During the 2023–2024 academic year, a self‐developed eight‐item questionnaire, together with a single overall‐satisfaction item, was administered to 366 final‐year students in 3‐year associate degree nursing programmes at two clinical teaching bases in Chongqing, China; 318 valid responses (86.9%) were analysed. Analyses comprised descriptive statistics, between‐centre comparison, effect‐size estimation and content analysis of open‐ended suggestions.

**Results:**

About two‐thirds of students reported active participation in class discussions (64%), whereas far fewer joined extracurricular academic activities (31%) or a study group (28%). About half considered teaching methods diverse (53%) or felt encouraged to express their opinions (50%). Written suggestions centred on interdisciplinary case teaching, emergency care training, and more diverse assessment. No item differed significantly between the two centres.

**Conclusions:**

Participation clustered in timetabled sessions and appeared to fall sharply for activities requiring students' own time, and the interactive quality of teaching was rated below its content. Routinely collecting and acting on student feedback may offer clinical teaching bases a practical way to address both.

**Implications for the Profession:**

Participation was consistently higher for timetabled activities, pointing to scheduling as a lever educators can act on, and fewer than one‐third of students volunteered a concrete suggestion. Nurse educators and clinical teaching administrators may draw on these student‐derived priorities when adjusting curricula, training preceptors and designing feedback mechanisms.

**Impact (Addressing):**

- What problem did the study address?
  ○ Evidence on students' clinical teaching participation was limited.
- What were the main findings?
  ○ Participation was high for scheduled activities, lower for voluntary ones.
- Where and on whom will the research have an impact?
  ○ Findings may guide scheduling and preceptor training at teaching bases.

**Reporting Method:**

Reported in accordance with the Strengthening the Reporting of Observational Studies in Epidemiology (STROBE) checklist for cross‐sectional studies.

**Patient or Public Contribution:**

No patient or public contribution.

## 1 Introduction

In China, ‘clinical teaching bases’ are accredited teaching hospitals that partner with a nursing school and take responsibility for students' clinical education. During the final‐year internship, students rotate through multiple departments at the base and concurrently take theoretical courses (such as nursing management and professional development) delivered on site by hospital‐based educators. Bedside teaching and classroom instruction therefore run in parallel within the same hospital setting, making the base a pivotal environment for the transition from theoretical knowledge to clinical skill (Immonen et al. 2019; Carless‐Kane and Nowell 2023). Throughout this paper, ‘class discussions and activities’ refers to these on‐site theoretical course sessions.

Student participation, also termed student engagement, refers to the degree to which students actively take part in learning activities. The present study operationalizes it as three behaviours: taking part in class discussions and activities, belonging to a collaborative study group, and joining extracurricular academic activities. Each is a recognized correlate of learning: active participation is associated with better comprehension and retention (Deslauriers et al. 2019), collaborative learning with higher academic achievement (Zhang and Maconochie 2022), and extracurricular academic participation with satisfaction and academic performance (Leksuwankun et al. 2023). In nursing specifically, students' appraisal of the clinical learning environment and of teaching quality is associated with their intention to work as a nurse after graduation (Zhang et al. 2022).

A third, closely related element is student feedback, or ‘student voice’: students' appraisal of courses and teaching together with their suggestions for improvement. In nursing education, such evaluations and qualitative suggestions are treated as indicators of the quality of the educational experience (Cant et al. 2023) and have been used to strengthen curriculum relevance and practice readiness (Calma et al. 2019). Most existing evidence on engagement and satisfaction, however, comes from university classrooms or Western educational settings (Deslauriers et al. 2019; Cant et al. 2023); little is known about the participation, teaching perceptions and improvement priorities of nursing students during internships at Chinese clinical teaching bases. At the two bases studied here, teaching quality had previously been judged mainly through supervisory inspection and instructor assessment, with no routine collection of structured student feedback. This matters for practice as much as for education: clinical teaching bases shape the competence of the incoming nursing workforce, so the weaknesses students identify point to where the preparation of new nurses might be strengthened. Nurse educators and clinical teaching administrators are positioned to act on such evidence.

This study therefore surveyed nursing students at two clinical teaching bases in order to describe their participation, their perceptions of teaching, and the improvements they prioritize. Three research questions were addressed:

Research Question 1 (RQ1) What are the current levels of participation among nursing students at clinical teaching bases?

Research Question 2 (RQ2) How do students perceive the effectiveness of current nursing teaching methods and content?

Research Question 3 (RQ3) Based on student feedback, what specific improvements can nursing education programmes make?

## 2 Methods

### 2.1 Study Design and Ethical Considerations

This was a cross‐sectional, descriptive questionnaire survey, reported in accordance with the STROBE checklist for cross‐sectional studies (von Elm et al. 2007). The first page of the questionnaire set out the purpose of the study, its voluntary nature and its anonymity. The study protocol was approved by the ethics committees of the participating institutions. Because the survey was fully anonymous, the committees waived the requirement for written informed consent; completion of the questionnaire was taken as consent. Participants' anonymity was preserved and no personally identifying data were collected. The study was conducted in accordance with the Declaration of Helsinki.

### 2.2 Participants and Sampling

The study was conducted at two tertiary teaching hospitals in Chongqing, China, approximately 150 km apart, both serving as clinical teaching bases for nursing education programmes. Eligible participants were full‐time final‐year students in three‐year associate degree (diploma) nursing programmes who were completing their final clinical internship semester, during which they rotated through multiple specialty departments and concurrently took theoretical courses. Students repeating the year and those who had taken part in pilot testing of the questionnaire were excluded. A census approach was used: every eligible student at both bases was invited to take part. The questionnaire was administered on site in group sessions during the final month of the internship semester (May 2024) and collected immediately on completion; by that point students had finished most of their rotations and could comment on their educational experience from an informed position.

### 2.3 Questionnaire Development and Validation

An initial item pool was generated from a literature review and expert consultation, then refined against five criteria: relevance, actionability, applicability across settings, ease of response and absence of redundancy. The final instrument began with a brief demographic section (sex and age) and continued with eight core items in two parts: student participation (3 dichotomous items) and teaching perception (5 items, the last of which carried an open‐ended field for improvement suggestions). A ninth item, answered separately, recorded overall satisfaction from 1 (very dissatisfied) to 5 (very satisfied). All items are listed in Table 1. Development and validation procedures, and the instrument's performance in the analytic sample, are reported in Table S1.

**TABLE 1:** Questionnaire structure and content.

No.	Item	Response options
*Part 1. Student participation*		
1	Do you actively participate in class discussions and activities?	□ Yes □ No
2	Are you a member of any study groups or academic teams?	□ Yes □ No
3	Do you participate in extracurricular academic activities (e.g., lectures and competitions)?	□ Yes □ No
*Part 2. Teaching perception*		
4	Is the course content comprehensive and aligned with the characteristics of the nursing discipline?	□ Substantial □ Average □ Not substantial
5	Are diverse teaching methods (e.g., case studies and group discussions) employed during instruction?	□ Diverse □ Relatively singular
6	Do instructors actively encourage student participation and expression of opinions during teaching?	□ Yes □ No
7	Are you satisfied with the instructors' teaching quality and attitude?	□ Satisfied □ Average □ Not satisfied
8	Are you willing to provide suggestions for improving current teaching methods?^a^	□ Yes (please describe below) □ No
9	Overall, how satisfied are you with the teaching at this clinical teaching base?^b^	□ 1 □ 2 □ 3 □ 4 □ 5

^a^ Open‐ended field for improvement suggestions; the source of the qualitative data.

^b^ Single item outside the 8‐item categorical questionnaire; analysed as a continuous score; □ 1 (very dissatisfied) → □ 5 (very satisfied).

### 2.4 Data Analysis

Analyses were performed in IBM SPSS Statistics version 27.0 (IBM Corp., Armonk, NY, USA) and in R version 4.6.1 (R Foundation for Statistical Computing, Vienna, Austria) via RStudio. Frequencies, percentages, means, and standard deviations were computed for all items, with 95% confidence intervals (CIs) for proportions derived by the Wilson score method. Internal consistency was re‐examined in the analytic sample, using KR‐20 for the three dichotomous participation items and Cronbach's *α* for the four teaching‐perception rating items (Items 4–7). Associations between items were summarized with Pearson correlation coefficients. As a secondary, exploratory analysis, the two centres were compared using chi‐square tests across the full response categories of each item, with Cohen's *h* as the effect size. Item 9 (overall satisfaction) was compared with an independent‐samples *t*‐test and Cohen's *d*. For the three items with more than two response levels (Items 4, 7 and 8), Cohen's *h* contrasts the most positive category against the remaining categories combined, whereas the corresponding chi‐square test uses the full set of response categories. No adjustment was made for multiple comparisons. A two‐tailed *p* < 0.05 was considered statistically significant. Free‐text responses to Item 8 were analysed by conventional content analysis, with themes derived inductively; themes were not mutually exclusive, so their percentages sum to more than 100%. Quantitative and qualitative analyses were each performed independently by two researchers, with disagreements resolved by a third.

## 3 Results

### 3.1 Participants

All 366 distributed questionnaires were returned. Forty‐eight were returned entirely blank (students who chose not to respond, treated as declining participation) and were excluded, leaving 318 fully completed questionnaires for analysis (valid response rate 86.9%; Centre 1, *n* = 156; Centre 2, *n* = 162). All participants were female, with a mean age of 20.53 years (SD 0.84), and the two centres did not differ in age (Table 2).

**TABLE 2:** Student engagement and perceptions of teaching among nursing students at two clinical teaching bases (*N* = 318).

Item	Response	Overall (*N* = 318) *n* (%, 95% CI)	Centre 1 (*n* = 156) *n* (%, 95% CI)	Centre 2 (*n* = 162) *n* (%, 95% CI)	*p*
*Participant characteristics*					
1. Sex	Female	318 (100%)	156 (100%)	162 (100%)	—
2. Age, years	Mean (SD)	20.53 (0.84)	20.56 (0.86)	20.50 (0.83)	0.499^b^
*Part 1. Student participation*					
1. Active participation in class discussions	Yes	204 (64.2%, 58.7–69.2)	99 (63.5%, 55.7–70.6)	105 (64.8%, 57.2–71.7)	0.801^a^
	No	114 (35.8%, 30.8–41.3)	57 (36.5%, 29.4–44.3)	57 (35.2%, 28.3–42.8)	
2. Joined study groups or academic teams	Yes	89 (28.0%, 23.3–33.2)	42 (26.9%, 20.6–34.4)	47 (29.0%, 22.6–36.4)	0.678^a^
	No	229 (72.0%, 66.8–76.7)	114 (73.1%, 65.6–79.4)	115 (71.0%, 63.6–77.4)	
3. Participated in extracurricular academic activities	Yes	99 (31.1%, 26.3–36.4)	47 (30.1%, 23.5–37.7)	52 (32.1%, 25.4–39.6)	0.704^a^
	No	219 (68.9%, 63.6–73.7)	109 (69.9%, 62.3–76.5)	110 (67.9%, 60.4–74.6)	
*Part 2. Teaching perception*					
4. Course content is comprehensive and aligns with subject characteristics	Substantial	218 (68.6%, 63.3–73.4)	107 (68.6%, 60.9–75.4)	111 (68.5%, 61.0–75.2)	0.614^a^
	Average	66 (20.8%, 16.7–25.5)	30 (19.2%, 13.8–26.1)	36 (22.2%, 16.5–29.2)	
	Not substantial	34 (10.7%, 7.8–14.6)	19 (12.2%, 7.9–18.2)	15 (9.3%, 5.7–14.7)	
5. Diverse teaching methods used during instruction	Diverse	168 (52.8%, 47.3–58.3)	84 (53.8%, 46.0–61.5)	84 (51.9%, 44.2–59.4)	0.722^a^
	Relatively singular	150 (47.2%, 41.7–52.7)	72 (46.2%, 38.5–54.0)	78 (48.1%, 40.6–55.8)	
6. Instructors actively encourage student participation and expression	Yes	159 (50.0%, 44.5–55.5)	81 (51.9%, 44.1–59.6)	78 (48.1%, 40.6–55.8)	0.501^a^
	No	159 (50.0%, 44.5–55.5)	75 (48.1%, 40.4–55.9)	84 (51.9%, 44.2–59.4)	
7. Satisfaction with instructor's teaching quality and attitude	Satisfied	205 (64.5%, 59.1–69.5)	106 (67.9%, 60.3–74.8)	99 (61.1%, 53.4–68.3)	0.441^a^
	Average	91 (28.6%, 23.9–33.8)	40 (25.6%, 19.4–33.0)	51 (31.5%, 24.8–39.0)	
	Not satisfied	22 (6.9%, 4.6–10.3)	10 (6.4%, 3.5–11.4)	12 (7.4%, 4.3–12.5)	
8. Willingness to provide suggestions for improving teaching methods	Willing, suggestion provided	98 (30.8%, 26.0–36.1)	45 (28.8%, 22.3–36.4)	53 (32.7%, 26.0–40.3)	0.164^a^
	Willing, no suggestion	96 (30.2%, 25.4–35.4)	42 (26.9%, 20.6–34.4)	54 (33.3%, 26.5–40.9)	
	Not willing	124 (39.0%, 33.8–44.5)	69 (44.2%, 36.7–52.1)	55 (34.0%, 27.1–41.5)	
9. Overall satisfaction with teaching at the clinical teaching base	Mean (SD)	3.43 (0.95)	3.53 (0.93)	3.34 (0.97)	0.072^b^

*Note:* Data are presented as *n* (%, 95% CI) unless otherwise indicated; CIs use the Wilson score method. Item 8 was recorded as a yes/no item, and the two willing categories reflect whether a teaching‐related suggestion was provided.

^a^ Pearson's chi‐square test.

^b^ Independent‐samples *t*‐test.

### 3.2 Participation and Perceptions of Teaching (RQ1, RQ2)

Internal consistency was acceptable for both subscales in this sample (participation, Items 1–3: KR‐20 = 0.71; teaching perception, Items 4–7: Cronbach's *α* = 0.75). Participation was highest for timetabled classroom discussions (204/318, 64.2%, 95% CI 58.7–69.2) and about half that level for extracurricular academic activities (99/318, 31.1%, 95% CI 26.3–36.4) and self‐organized study groups (89/318, 28.0%, 95% CI 23.3–33.2). Study‐group membership and extracurricular participation were moderately correlated (*r* = 0.56). Perceptions of teaching were less uniform. Course content (218/318, 68.6%, 95% CI 63.3–73.4) and overall teaching quality (205/318, 64.5%, 95% CI 59.1–69.5) were rated positively by roughly two‐thirds of students, whereas only about half considered teaching methods diverse (168/318, 52.8%, 95% CI 47.3–58.3) or felt encouraged to express their views (159/318, 50.0%, 95% CI 44.5–55.5); the remainder gave neutral or negative ratings. Overall satisfaction with teaching at the base averaged 3.43 (SD 0.95) on the 5‐point scale (Table 2).

### 3.3 Student Feedback and Improvement Suggestions (RQ3)

Engagement with the feedback process itself was limited. Of the 318 students, 194 (61.0%, 95% CI 55.5–66.2) were willing to comment on teaching and 124 (39.0%, 95% CI 33.8–44.5) declined outright; among those willing, 96 (30.2% of the full sample, 95% CI 25.4–35.4) reported having no specific suggestion (Table 2). The remaining 98 students (50.5% of willing students; 30.8% of the full sample) provided at least one written suggestion. Content analysis identified five themes: interdisciplinary case teaching (28/98, 28.6%), emergency care training (26/98, 26.5%), diversified assessment methods (22/98, 22.4%), technology integration (18/98, 18.4%), and current‐affairs topics (15/98, 15.3%) (Figure 1). The 98 responses yielded 109 coded mentions (mean 1.1 themes per respondent), and 9 responses (9.2%) covered more than one theme.

**FIGURE 1:**
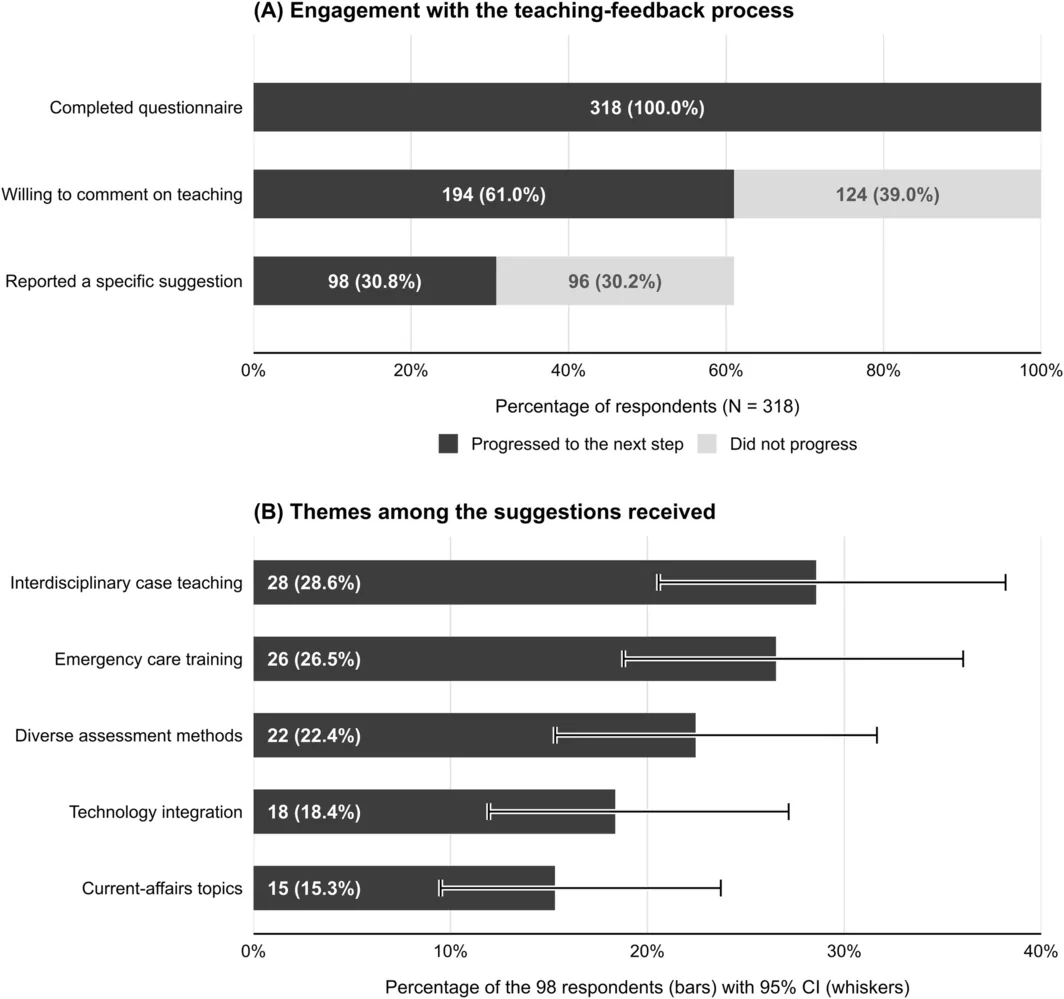
Engagement with the teaching‐feedback process (A) and themes among the 98 written suggestions (B).

### 3.4 Comparison Between the Two Centres (Exploratory)

No categorical item differed significantly between centres (all *p* > 0.05; Table 2), and all effect sizes for these items were negligible (|Cohen's *h*| ≤ 0.143; Table 3). Overall satisfaction came closest to a between‐centre difference (Cohen's *d* = 0.20), although its confidence interval included zero (Tables 2 and 3).

**TABLE 3:** Between‐centre differences and effect sizes for each survey item.

Comparison item	Centre 1	Centre 2	Difference^a^	Effect size	Interpretation
1. Active participation in class discussions	63.5	64.8	−1.4	−0.028^b^	Negligible
2. Joined study groups or academic teams	26.9	29.0	−2.1	−0.047^b^	Negligible
3. Participated in extracurricular academic activities	30.1	32.1	−2.0	−0.043^b^	Negligible
4. Course content substantial	68.6	68.5	+0.1	+0.002^b^	Negligible
5. Diverse teaching methods	53.8	51.9	+2.0	+0.040^b^	Negligible
6. Instructors encourage participation	51.9	48.1	+3.8	+0.076^b^	Negligible
7. Satisfied with teaching quality	67.9	61.1	+6.8	+0.143^b^	Negligible
8. Willing and provided a suggestion	28.8	32.7	−3.9	−0.084^b^	Negligible
9. Overall satisfaction, mean score	3.53	3.34	+0.19	+0.203^c^	Small

^a^ pp, percentage points (Centre 1 minus Centre 2; positive values indicate a higher proportion in Centre 1).

^b^ Items 1–8 are proportions, compared with Cohen's *h*; by convention 0.2, 0.5 and 0.8 denote small, medium and large effects, and |*h*| < 0.2 was regarded as negligible.

^c^ Item 9 is a mean score on a 5‐point scale, compared with Cohen's *d*; 95% CI −0.02 to 0.42.

## 4 Discussion

Three patterns emerged from this survey of 318 final‐year nursing interns at two clinical teaching bases. Participation appeared to be stratified more by how learning was scheduled than by its academic content; teaching was rated more favourably for course content and overall quality than for its interactive dimensions; and most students did not convert a general willingness to comment into a specific suggestion.

### 4.1 Stratification of Student Participation

The divide in these data appears to lie less between academic and non‐academic activity than between scheduled and unscheduled learning. Classroom discussion is timetabled into the working day, whereas study groups and extracurricular academic activities both require students to commit time outside their rostered hours, and participation in both was roughly half as high. One possible explanation, not tested here, is that lower participation reflects the constraints of internship scheduling. Interns are dispersed across departments, work staggered shifts, and move to a new department every few weeks, which makes stable peer study groups hard to sustain. The moderate correlation between study‐group membership and extracurricular participation (*r* = 0.56) is consistent with the two behaviours being limited by a shared constraint rather than varying independently. This matters because peer collaborative learning develops the communication and teamwork skills that are core competencies for clinical nursing practice (Gao et al. 2024); where bases leave collaborative learning entirely to students to organize, that development may be left largely to chance.

### 4.2 Teaching Perceptions: Content Versus Delivery

Students rated course content and teaching quality more positively than the two interactive dimensions of teaching (method diversity and encouragement to speak up), on which close to half gave a neutral or negative rating. Interaction quality, alongside instructors' subject expertise and communication skills, is an established contributor to nursing students' satisfaction (Zhang et al. 2022). One plausible explanation for the gap is that clinical instructors' primary responsibility is patient care, leaving limited time to prepare for teaching. This would be consistent with reports that clinical preceptors are often inadequately prepared for the teaching component of their role (Al‐Daken et al. 2024; Good 2021). The constraint appears to lie less in what is taught than in how it is delivered.

### 4.3 Willingness to Give Feedback and the Content of Suggestions

Using student feedback to improve teaching presupposes that students are willing to evaluate; here, 39.0% declined to comment at all and a further 30.2% had no specific suggestion. Existing work notes that nursing curriculum evaluation often lacks meaningful feedback (Reis et al. 2024), that students' teaching evaluations are shaped by contextual factors (Tomas et al. 2022), and that willingness and comfort with evaluation vary considerably, including in peer‐feedback settings (Wong and Shorey 2022). The present findings extend this: before the quality of feedback can be debated, a substantial proportion of students have not entered the feedback process at all. Several features of the internship setting could contribute: doubts about anonymity, instructors who also assess interns' performance, no visible follow‐up on earlier comments, or unfamiliarity with how to evaluate teaching. A cross‐sectional design cannot distinguish between them.

Among students who did comment, interdisciplinary case teaching and emergency care training were the two most frequent themes, both consistent with existing evidence: interdisciplinary case teaching supports the integration of knowledge across disciplines into comprehensive care plans (Jung et al. 2020), and simulation‐based emergency training improves nursing students' knowledge and practical skills (Kabuk et al. 2024). Technology integration was raised less often, which may reflect the variable receptivity to multimedia and technology‐based teaching tools reported elsewhere (Ongor and Uslusoy 2023).

Across all three findings, students' learning behaviour appeared to track the institutional scaffolding available to them: where the school or hospital organized the activity, participation was substantially higher than where it was left to students themselves. Raising engagement may therefore depend less on encouraging students individually than on how teaching and feedback are organized. Documenting them together among interns at Chinese clinical teaching bases captures what a single engagement score would not: the gap between organized and self‐directed participation, and the proportion of students who opt out of feedback entirely.

### 4.4 Comparability of Results Between the Two Centres

The between‐centre comparison was exploratory; it was intended to justify pooling the two datasets for descriptive purposes, not as an equivalence test. The similarity of findings may reflect shared institutional features, such as a common national curriculum framework, comparable accreditation standards and similar student populations. The uniformly small effect sizes (Table 3) are compatible with standardized practice supporting consistent educational quality across sites, although identifying which specific mechanisms are responsible, such as faculty development, shared teaching resources, or routine quality assurance, would require a study designed for that purpose.

### 4.5 Limitations

Several limitations should be noted. First, all participants were female associate degree students recruited from two centres in one region during a single academic year, which limits generalizability to other educational contexts. Second, the study relied on voluntary participation and self‐report, so self‐selection and response bias cannot be excluded. Third, the scheduling explanation offered for lower participation in self‐organized activities remains untested; the study was also not designed to test between‐centre differences, and the qualitative sub‐sample (*n* = 98) was small. Fourth, the dichotomous and holistic item formats limited the specificity of the information obtained, with most actionable detail coming from the open‐ended responses. Finally, the questionnaire was developed on small preparatory samples (*n* = 20 at each stage), which is below conventional requirements for factor analysis and limits the precision of the psychometric estimates.

### 4.6 Implications

Three practical directions may follow for clinical teaching bases. First, collaborative learning could be timetabled into the internship itself (e.g., a fixed weekly discussion slot), so that it no longer depends on students coordinating around their own rotation schedules. Second, the feedback mechanism could be redesigned to lower the barrier to comment: separating, as far as is practical, the staff who collect feedback from those who assess interns' performance, and issuing a written response after each round so that students can see where their input leads. Third, because the interactive dimension of teaching was rated below content delivery, preceptor training could target participatory skills specifically, such as structured questioning, case‐based discussion and techniques for eliciting student contributions.

Future research could follow participation longitudinally across an internship and examine its association with licensure performance and workforce retention, and could pair questionnaires with semi‐structured interviews to establish why students withhold feedback. This is a question that a cross‐sectional design can raise but not answer.

## 5 Conclusion

Among final‐year nursing students at two clinical teaching bases, participation was moderate in timetabled classroom activities but substantially lower in extracurricular and peer‐organized learning, and the interactive quality of teaching was rated below its content. The interpretation offered for both patterns is descriptive and hypothesis‐generating: the organization of teaching may be a more tractable target for improvement than student motivation. Building routine, responsive collection of student feedback into curriculum design may offer nursing programmes one way to identify such targets and could help students prepare for the complexity of clinical work.

## Author Contributions

**Hao Zhang:** formal analysis, validation, funding acquisition, conceptualization, writing – review and editing, writing – original draft, visualization, methodology. **Yuan Mei:** data curation, investigation, writing – review and editing, resources, methodology. **Chun‐ling Zhang:** conceptualization, methodology, writing – original draft, writing – review and editing. **Jie Liu:** data curation, investigation, writing – review and editing, resources, methodology. **Xu‐jue Ma:** validation, writing – review and editing, investigation, methodology.

## Funding

This work was supported by the Teaching and Research Project of Chongqing Medical and Pharmaceutical Vocational Education Group (grant number CQZJ202606).

## Disclosure

Statistical Declaration: The authors have checked to make sure that this submission conforms, as applicable, to the Journal's statistical guidelines. There is a statistician on the author team: Hao Zhang. The authors affirm that the methods used in the data analyses are suitably applied to their data within their study design and context, and that the statistical findings have been implemented and interpreted correctly. The authors agree to take responsibility for ensuring that the choice of statistical approach is appropriate and is conducted and interpreted correctly, as a condition of submission to the Journal. Full details of the statistical methods, including the software used, are reported in the Data Analysis section of the manuscript.

## Ethics Statement

This study received approval from the Institutional Review Board of Dianjiang People's Hospital of Chongqing (Approval number: DYLL‐KY‐2023‐012; date of approval: March 16, 2023). Due to the anonymous nature of the questionnaire, written consent was waived by the Ethics Committee; completion of the questionnaire was taken as consent. All procedures were conducted following the ethical guidelines established by the Declaration of Helsinki. Participants were ensured anonymity, and no personal data were collected throughout the research process.

## Conflicts of Interest

The authors declare no conflicts of interest.

## Supporting information


**Table S1:** Validity and reliability assessment of the nursing education questionnaire.

## Data Availability

The data that support the findings of this study are available from the corresponding author upon reasonable request.
