# Supplementary material for: Nursing Students' Participation and Perceptions of Teaching Effectiveness at Clinical Teaching Bases: A Cross‐Sectional Survey
Source: Nurs Open. 2026 Oct 6;13(10):e70824. doi: 10.1002/nop2.70824 (PMC13639934; doi:10.1002/nop2.70824)
Supplement: Supplementary file 1 [file NOP2-13-e70824-s001.docx]

**Supplementary Table S1. Validity and Reliability Assessment of the Nursing Education Questionnaire**

| **Validation Type** | **Method** | **Participants** | **Metrics & Results** | **Outcome** |
| --- | --- | --- | --- | --- |
| **Questionnaire development (pre-survey)** | | | | |
| Content Validity | Expert Panel Review | 5 experts in nursing education and clinical practice | • Content Validity Index (CVI) • Items with CVI < 0.80 were revised/removed  Results by domain: 1. Student Participation: CVI = 0.92 2. Teaching Methods: CVI = 0.88 3. Course Content: CVI = 0.85 4. Student Feedback: CVI = 0.83 | • 3 items revised • 2 items removed |
| Face Validity | Pilot Testing | 20 nursing students^a^ | Feedback collected on: 1. Item clarity (rated 1-5) 2. Relevance (rated 1-5) 3. Completion time  Results: • Average clarity rating: 4.2/5 • Average relevance rating: 4.5/5 • Mean completion time: 12 minutes | • 4 items reworded for clarity • Response options simplified for 2 items • Instructions enhanced |
| Construct Validity | Exploratory Factor Analysis | Same pilot sample (n = 20)^a^ | • Factor loadings criterion: > 0.4 • KMO = 0.82 • Bartlett's Test: p < 0.001  Two-factor solution: 1. Student participation (3 items) 2. Teaching perception (5 items) | • 2 items eliminated due to low factor loadings (<0.4) • Final questionnaire: 8 items |
| Reliability | Test–retest (2-week interval) | Separate sample (n = 20)^b^ | • Pearson’s r = 0.86 | Scores were stable across the 2-week interval |
| **Psychometric performance in the analytic sample** | | | | |
| Reliability | Internal Consistency | Final sample (N = 318) | • KR-20 (Student Participation subscale, Items 1–3) = 0.714 • Cronbach’s α (Teaching  Perception subscale, Items 4–7; the open-ended suggestion item is excluded from scoring) = 0.750 | Both subscales exceeded the 0.70 threshold |

*Note.* CVI, content validity index; KMO, Kaiser–Meyer–Olkin measure of sampling adequacy. A loading of 0.40 was the criterion for assigning an item to a factor. Total-scale α is not reported because the subscales are multidimensional and use mixed item formats. ^a^ The same 20 pilot participants completed both procedures. ^b^ A separate group of 20 students. Neither ^a^ nor ^b^ contributed to the final analytic sample.
